# The Art of Coaching: Addressing Mobile Technology Integration in a Complex Hospital School Environment

**DOI:** 10.5334/cie.6

**Published:** 2020-06-16

**Authors:** Dorit Maor, Katherine Mitchem, Rachel Drewry

**Affiliations:** 1 Murdoch University, AU; 2 California University of Pennsylvania, US

**Keywords:** coaching, professional development, hospital schoolteachers, mobile technology integration

## Abstract

This qualitative study examined the effects of a coaching program as part of a professional development effort to support integration of mobile technologies in a hospital school setting. The professional development consisted of two components: (a) the researchers introduced pedagogical models for incorporating mobile technology in education and (b) a technological expert (the iCoach) provided one-on-one and small-group personalized coaching. After studying the contextual challenges and teachers’ needs over a period of 12 months, an effective coaching model emerged. This was a personalized model geared to each teacher and tailored to the unique features of this hospital context. An integral part of the model was a reflection strategy, which encouraged teachers to question their teaching with the purposeful use of technology. Data collection involved teachers’ reflections, iCoach reflections, and semi-structured interviews. Many themes emerged from the data analysis; the two main ones were the uniqueness of the setting and the multiplicity of demands on the teachers and the iCoach. The study developed a set of guidelines to help teachers use technology in an integrated pedagogical way.

## Introduction

Providing appropriate education to children unable to attend regular school is challenging. In many countries, some students with emotional or health challenges receive educational services in a variety of settings, including alternative schools, residential psychiatric placements, hospitals, and students’ homes for short and long periods of time (Halfon, Houtrow, Larson, & Newacheck, 2012; Hopkins, 2015). The educational progress and social and emotional wellbeing of hospitalized students can suffer because of displacement from schools, friends, and families (A’Bear, 2014; Wakefield et al., 2010).

In Australia, hospital schools are typically small schools situated in regional hospitals catering to school-age children and staffed by teachers under the jurisdiction of the local Department of Education. These hospital schoolteachers can help students using a range of educational delivery options to maintain their learning and reduce the isolation and anxiety that disrupted schooling may cause.

The aim of this research was to investigate a personalized coaching program developed in an Australian hospital to meet the unique needs of hospital schoolteachers. Specifically, the purpose of the program was to provide professional development to these teachers by introducing mobile technology. To help teachers with the complex role of teaching children in the unique environment of a hospital school, the leadership group decided to embark on an educational journey of integrating mobile technologies to improve teaching. To make the process effective for the teachers, professional development (PD) was offered consisting of two components: (a) the introduction of pedagogical models for incorporating mobile technology in education and (b) one-on-one and small-group personalized coaching provided by a technological expert (the iCoach).

The pedagogical component was delivered through a series of events across the year, and included at least one full day of training per term for all the hospital teachers (McCarthy, Maor, & McConney, 2017). This paper focuses on the unique coaching model that the external expert delivered through a personalized program. We refer to the external expert as *iCoach* as this was the label bestowed by teachers in the study. The iCoach was a specialist in the use of mobile technologies in teaching and learning and a special needs educator who guided and supported the teachers. According to Daloz (2012), coaching is defined as providing personal opportunities for applicable, meaningful, and substantial experiences, along with ongoing support, constructive feedback, and continuous monitoring from an expert.

The challenges of the unique context of the hospital school and the diversity of students in terms of age, background, and ability made the personalized coaching model highly relevant to the teachers’ professional needs. Therefore, the goal of the iCoach was to provide personalized learning opportunities to improve the participating teachers’ technological and pedagogical knowledge and ability to better integrate mobile technologies into their teaching. The external iCoach was guided by contemporary theoretical frameworks, including Technology Pedagogy and Content Knowledge (TPACK; Koehler & Mishra, 2009; Mishra & Kereluik, 2011), Substitution, Augmentation, Modification, and Redefinition (SAMR; Puentedura 2012), along with individualized job-embedded coaching and personal reflection (Yendol-Hoppey & Dana, 2010). The coach’s role was to work through cycles of coaching with participant teachers. Each cycle was based on meeting teacher needs, assisting with the implementation of new technology, and reflecting on the use of mobile technologies with the hospitalized students.

Participating teachers volunteered to take part in the research. Any teacher could elect to be part of the personalized coaching, and the iterative process allowed opportunities for some teachers to participate in coaching more than once and accommodated their varying availability and schedules. The conceptual models (TPACK, SAMR) helped facilitate the coaching process for each teacher, including reflecting on their teaching and the use of the technology.

The mobile technologies that were used by the teachers included portable devices (tablets, iPads, or smart phones) connected to the Internet, which could be used by teachers and students anytime, anywhere in the hospital school classroom or at the bedside of the sick child. These devices were particularly appropriate for communication with parents, collaboration with teachers at the enrolled schools, and access to a wide range of curricula knowledge and skills for meeting the complex and challenging needs of diverse student populations at multiple locations in hospital. Our definition of mobile technologies also extended to the educational applications that were used by the teachers as required.

In recent studies by Hutchison and Woodward (2018) and Thoma, Hutchinson, Johnson, Johnson, and Stromer (2017), students of teachers who had completed training based on a Technology Integration Planning Cycle (Thoma et al., p. 167) performed significantly better on digital literacy assessments. This coaching cycle was conducted in the teaching context on an ongoing basis and provided access to instructional coaches and learning communities as well as digital resources. In the hospital school context outlined in this paper, similar components were provided to the teachers. That is, the iCoach gave them ongoing and intense technological and pedagogical support and, as a result, this small community of teachers gradually became highly trained in and confident about integrating technology into their pedagogy.

## Literature Review

### Teaching and Learning in Hospital School Contexts

The adverse outcomes of disruptive schooling and the social and emotional impact of hospitalization on children have been widely discussed (Capurso & Dennis, 2017; Hopkins, Green, Henry, Edwards, & Wong, 2014). However, despite the recognition that mobile technologies have the potential to transform the teaching and learning environments in hospital schools (Botha, Batchelor, Traxler, de Waard, & Herselman, 2012; Owen, 2015), the research literature reveals that integration of mobile technologies into hospital schools is limited. This transformation can be achieved by engaging students to participate and collaborate with other learners, online, or face-to-face (Fullan & Langworthy, 2013; Hattie & Anderman, 2013).

Teachers in hospital schools face unique challenges. They must be prepared to teach a wide range of topics to a wide range of students, from varying jurisdictions, with multiple complex needs, and varying levels of motivation. In addition, hospital schoolteachers work in a number of diverse settings and different locations on any given day. Children requiring hospitalization frequently, or for significant periods of time, tend to experience trauma not only physically, but also emotionally and socially (Brokstein, Cohen, & Walco 2002; Bush & Simonian, 2002). In addition to limited contact with families and the disrupted routine of school life, hospitalized children must accept the restrictions of their new environment (Maor & Mitchem, 2015; McCarthy, Maor, & McConney, 2019). Prolonged absence from school can cause severe barriers to learning (Martinez & Ercikan, 2009) and, in the case of students who are required to complete major assessments, high anxiety and stress.

Hospital teachers’ communication and collaboration with parents and other educators and outside school administrators is critical for successful transition (Franck, Gay, & Rubin, 2013). Mobile learning has emerged from the broader field of e-learning (Oakley, Pegrum, Faulkner, & Striepe, 2012; Oakley, 2017) as a preferred way of learning and communication. Thus, the adoption of devices such as iPads and tablets has had significant implications for teaching and learning as students slip in and out of formal learning spaces (Keane, Lang, & Pilgrim, 2012; Oakley et al., 2012), removing constraints of time and place (Traxler, 2010).

### Professional Development and Mobile Technology Integration

Although mobile technologies can positively support student engagement and learning with new pedagogical practices (Ally, Grimus, & Ebner, 2014), teachers often struggle when integrating technology (Kopcha, 2012). For example, many teachers need help selecting effective pedagogical strategies for using technology and are unaware of mobile technology’s personal, administrative, and teaching benefits (Owen, 2015).

Much is known about professional development’s requisite features of effectiveness, including actively engaging teachers in learning (Pierson & Borthwick, 2010); developing collaborative communities (Lofthouse, 2019; Sindelar & Brownell, 2001); providing expert support (Zorfass & Rivero, 2005); and focusing on interventions that are practical, meet specific needs, and are aligned with local contexts (McLesky & Waldron, 2004). A good relationship between coaches and teachers is also important to the success of the coaching process. This is often dependent on building positive relationships and effective communication through collaboration and coach guidance (Mitchell, Kwok, & Husten, 2019; Robertson, Ford-Connors, Frahm, Bock, & Paratore, 2019). Finally, many researchers have concluded that in order for teachers to use and integrate technology meaningfully, they must be guided by appropriate theoretical frameworks (Capurso & Dennis, 2017; Koehler & Mishra, 2009; Mishra & Kereluik, 2011; Puentedura, 2012) with individualized job-embedded coaching (Yendol-Hoppey & Dana, 2010).

### Coaching to Facilitate Integration of Technology

The research literature emphasizes the importance of coaching in professional development programs (Beglau et al., 2011; Blachowicz, Obrochta, & Fogelberg, 2005; Ehsanipour & Zaccarelli, 2017; Kraft, Blazar, & Hogan, 2016; Robertson et al., 2019), primarily because information and professional guidance can be tailored to a teacher’s knowledge, skills, and specific classroom circumstances (Powell & Diamond, 2013). Coaching can be central or a supplement to professional development. The key is a sustained collaborative relationship between the coach and teacher. Illustrating the importance of collaboration in coaching, Beglau et al. (2011) reported on the role of coaching, combined with professional learning communities, to facilitate effective use of technology in teaching and learning. In their study, teachers who received coaching in the use of technology developed confidence to support students in technology-rich environments that facilitated student learning (Beglau et al., 2011).

Coaching is particularly suited to situations where high levels of individualization are necessary, as is the case in hospital schools. Coaching can be customized to local contexts and can be used to promote collaboration and sharing of knowledge (Christensen et al., 2018; Hutchinson & Woodward, 2018; Montebello, 2017; Robertson et al., 2019). Coaching has specific advantages over other forms of professional development. For example, support by the coach can be provided in the context of the teacher’s practice at the point of need, and often integrates collective participation in its design, building expertise through collaboration (Ehsanipour & Zaccarelli, 2017; Garet, Porter, Desimone, Birman, & Yoon, 2001). Further, it can be offered on an ongoing basis, countering a common criticism that professional development is too short and lacks follow-up (Coburn, 2004; Tyack & Cuban, 1995).

According to Powell and Diamond (2013), the specific implementation of the individualized practices that comprise coaching has not been well defined and documenting coaching’s effectiveness would add to existing research. The present study was conducted to start filling this gap in the literature.

## Method

### Research Context

The role of the iCoach was to guide a group of four or five teachers/educational assistants for four weeks at a time. Staff members participated in several individual sessions with the iCoach, as well as in small-group sessions. The iCoach facilitated learning by acknowledging the difficulties and challenges that emerged throughout the process, rather than providing a predetermined professional development program.

The coaching block of four weeks ensured that most of the staff had access to the iCoach within a school year; some staff members engaged in several coaching cycles. The trajectory produced an iterative process that allowed the progressive development of skills and knowledge and was tailored for individual needs. The iCoach worked closely with the research team and continued to encourage the use of the TPACK and SAMR pedagogical models that were introduced at the PD school days to increase the teachers’ understanding of how to best use the mobile technologies and applications (Apps) in their teaching.

During the first session, the iCoach shadowed the teachers, observing how they worked with the students, assessing their needs with the goal of maintaining a narrow focus so that the teachers did not feel they had to use technology for everything. The teachers expressed multiple needs, including exploring creative applications, support for communication, and using Apps to replace worksheets.

In this first session the iCoach supported the teachers in setting goals for the coaching. For the next three sessions, the iCoach used a mini action reflection process in which the teachers identified their needs, decided on a solution with the iCoach, implemented the Apps suggested, or used other pedagogical solutions. In the following week, the iCoach and the individual teachers reflected on what worked and what didn’t work. The teachers were asked to record their thoughts, and in the following session they discussed their reflections and replanned for the next session. This mini action research cycle from one week to the next enabled teachers’ learning, implementation, and reflection on a weekly basis. In line with the findings of Kereluik, Mishra, Fahnoe, and Terry (2013), the iCoach’s reflection cycle, in turn, provided a deeper understanding of the complex world of hospital schoolteachers and their pedagogical and technological needs.

Initially, the iCoach selected Apps for the teachers. The unique situation of working with individual teachers across different age groups and different subjects at different times of the year proved challenging, particularly when selecting appropriate Apps for use with students. However, soon it became apparent that teachers were taking ownership and began to select and download Apps based on their revised goals.

In relation to the pedagogical models, the iCoach discussed these and then suggested the appropriate Apps for the particular pedagogical needs. Creative Apps are valuable and can be used more flexibly across learning areas. They can be differentiated for individual students and they encourage higher-order thinking. Gradually, within the four weeks of the first coaching cycle, teachers discussed the TPACK or SAMR models, and then selected and utilized the appropriate Apps for their pedagogical purpose (see Table 1). The iCoach’s trajectory was to build up the teachers’ pedagogical and technological knowledge and to increase their confidence in the use of new Apps.

**Table 1:** The Coaching Program Combining the Pedagogical Frameworks and Technology.

SAMR	

Substitution	e-books instead of hard-copy books
Augmentation	e-version of initial school self-assessment questionnaire to improve functionality
Modification	Students using iMovie to create and share a multimedia presentation of their learning
Redefinition	Using Skype to connect students with home school teacher/peers/learning program (e.g., Stitches in my Seat)
**TPACK**	

Polldaddy	This App already set up with a student self-assessment questionnaire.
Skills Road	www.skillsroad.com.au is a useful careers planning website. It has an in-depth Careers Quiz, an interactive Resume Builder, a Careers Guide and Jobs Board.
GarageBand	This App is used to create music and can be used in conjunction with iMovie. Students create their own music and can add it to their movie clip.
Ted Ed online lesson creator	This tool is used for building interactive lessons based on key Science topics. For example http://ed.ted.com/lessons/what-does-the-pancreas-do-emma-bryce#review
iMovie	This App is used for revision or consolidation of learning about cells.
Wordle	This App is used to create a visual graphic of topic key words.
Skwirk	This website is used an alternative to TED. The content is organized by year level and linked directly to the Australian curriculum.

### Participants

Two types of participants were involved in this study: teachers and the iCoach. Out of 75 teachers and teachers’ assistants in the hospital school, 29 volunteered to participate in the study and signed a consent form to be interviewed, but only 22 participated in the PD data collection. This attrition was due to changes in circumstances, such as service leave and illness.

#### Teachers

The teachers who volunteered to participate in the study were experienced, primary and secondary teachers who had an average of 26 years’ teaching experience and an average of 9 years’ teaching in hospital schools. More specifically, there were four secondary schoolteachers of history and social sciences, two in science and human biology, two in math, one in chemistry, and one in accounting. There were a further three primary schoolteachers who specialized in English, three general primary and early childhood education teachers, and four primary school teaching assistants. One English teacher was teaching in both primary and secondary areas. The 22 teachers who completed the coaching cycle taught a range of subjects, and most of them were female (82%).

#### iCoach

The digital technology iCoach was an external consultant employed by the hospital school in a part-time capacity. As an experienced teacher, she had previously engaged in digital transformation initiatives and 1:1 iPad programs in regular schools. At the time of the research, the iCoach had over 10 years’ teaching experience, including 7 years of integrating technology in teaching and developing her pedagogical understanding as an Apple Distinguished Educator (Apple Inc., 2019) during the previous 3 years. She also had experience as a special needs teacher, and at the time of the research she was completing her master’s degree in digital technology. The personal iCoach was selected to conduct individualized programs based on her special education and pedagogical-technological qualifications and her experience integrating teaching and technology.

### Research Design

The authors employed qualitative methods based on semi-structured interviews and the reflections of the teachers and the iCoach. Our emphasis was on issues that were raised by the teachers and the iCoach that could help develop a better PD program. In the results section, we present examples of the issues raised throughout the interviews by 20 teachers (see below). These excerpts are representative of the data.

### Procedure

#### Interviews

Teachers were interviewed individually; interviews lasted 30–60 minutes. Data were obtained in pre-professional development interviews about teachers’ technology integration and their needs prior to implementing coaching. Data obtained from post-professional development interviews identified what technology integration gaps and needs still existed and what coaching strategies were working. The number of pre- and post-interviews differ because some teachers were absent due to sick leave, long service leave, or retirement at the time of the post-interviews.

#### Teacher reflections

After each coaching session, teachers were encouraged to reflect on their teaching with technology for the given week. As the coaching progressed, the teachers discussed their reflections with the iCoach in the next session to reinforce the reflective process. They could record or write their reflections on an iPad. Most participants recorded audio, which was later transcribed.

#### iCoach reflections

Approximately once each month (a full coaching cycle), the iCoach recorded her own reflections, which were later transcribed.

#### iCoach interviews

The iCoach was interviewed twice for approximately 40 minutes each. In these interviews, she elaborated on technological and pedagogical aspects of the coaching and, in particular. the progress that individual teachers made.

The final information came from 20 pre- and post-interviews, 2 iCoach interviews, 6 iCoach reflections, and 26 teacher reflections. Two researchers analyzed the transcripts independently and noted the main ideas that were later classified using open-ended coding with key themes emerging from the process (Creswell, 2007). When disagreements occurred, the researchers met to discuss the codes and reach agreement to ensure stability (reliability) of coding (Creswell, 2007). Information from multiple methods and sources of data collection and analysis enabled triangulation of the themes.

Once we identified and agreed on our codes, we nominated emergent themes by independently sorting codes into categories, reflecting perceptions expressed by participants in all interviews about the use of mobile technologies in the hospital school. At this stage, all data were transferred to NVivo to facilitate further analysis. The researchers then met to compare and agree on the identified codes. We then revisited the transcript interviews several times to ensure that the code application was accurate and consistent by independently coding each interview (Maor & Mitchem, 2018).

One major theme was the uniqueness of the setting and the multiplicity of demands on teachers. In addition, a number of related subthemes provided a deeper understanding of the needs of teachers and students that subsequently shaped the process of coaching. Based on our qualitative analysis, the quotes presented in the Results section represent the variety of views from the teachers and the iCoach. Our aim was to identify the main issues expressed so that guiding principles and recommendations could be developed for future programs.

## Results

### The Main Contextual Challenges and Teacher Needs

The overarching theme that emerged from both the teachers and the iCoach was the uniqueness of the setting. Participants discussed the difficulties they faced as a result of the multiple challenges in the hospital. For example, a teacher referred to the diverse locations of children in the hospital, which amplified the need for mobile devices:

*I’ll have kids in the classroom, several in isolation, and some in day care, and then I need to be on other wards and all those places at different times. It would be good to know how to deal with that*. (Teacher-06-interview)

Teachers faced challenges in trying to meet the varying needs of many students often without being able to plan in advance. They constantly had to be able to problem solve spontaneously and argued that “it is a different day every day.” This was even more evident when there were short- and long-term stay hospitalized students as described by another teacher:

*… often we might only have them for one day: that provides one sort of issue. Another type of setting might be in Oncology where they are there all year. So, how do we provide for them and how do we make education as engaging and meaningful and practical*. (Teacher-03-interview)

The iCoach saw the promise of mobile devices for addressing various locations and the multiplicity of demands, while at the same time recognizing the challenges of successfully utilizing the technology in a hospital school environment:

*… for those kids who can’t get out of their beds; for those kids who are in isolation; for those kids who are just there on a one off visit; even for those kids who are there long term; technology can fit into all of that*. (iCoach-Reflection)

Another challenge was the need to help students stay up-to-date with their coursework from their home school to ease their transition once they were discharged. Since teachers did not know who would be on the wards each day or how able students would be to work, planning was challenging. Teachers often needed to identify resources on the fly. Students would say at the last minute: “I need to do this today.” (Teacher-05-Interview). In an effort to manage and address this issue, one teacher relied on her personal iPhone to prompt responses from her student; she described it as follows:

*But to me it is worth the dollar cost because it’s so convenient, it’s so instant. My computer, my desktop sits down in another part of the hospital where I don’t teach. My phone dings when I’ve got a message and there’s the work for the kids so I can give it to them immediately*. (Teacher-05-interview)

Issues were compounded by challenges that appear to plague all technology integration initiatives; namely, infrastructure problems. While these are not unique to the hospital setting, the challenges to teachers already facing multiple and complex demands can make integrating technology seem insurmountable and not worthwhile. The frustrations of teachers were evident in the following example of one specialist teacher who moved from ward to ward:

*I may have an idea for a student who wants to do a particular topic. I get there and can’t connect. Or, we’re not allowed to access YouTube or it will drop out. Connectivity is a major problem*. (Teacher-07-interview)

Health and safety concerns faced by the broader community are even more acute in the hospital setting. In discussing the integration of mobile technologies with their students, teachers noted their concerns for confidentiality with increased use of social media, the risk of greater exposure with the capability of technologies:

*… the issue of confidentiality is a bit of a concern because now all of these technologies can take photos and can get launched on Facebook. If kids are going to be taking other kids’ photos in hospital, whereas up until now that hasn’t been happening…* (Teacher-19-interview)

Mobile technologies have changed communication. Occasionally students were in direct contact with their enrolled schoolteachers. Whereas, in the past, the hospital schoolteachers used to be completely in charge of the student, now:

*… there’s a lot of contact from the student directly to their home [enrolled] teacher and you have to make sure you are part of that because you are the one who is facilitating their learning in tandem*. (Teacher-04-interview)

### Focus and Timing of Content of Coaching

Coming to terms with the dynamic nature of the hospital school environment was challenging for the iCoach who had years of experience as a special needs teacher and an instructional technology coach in a regular education setting. She was used to planning ahead, having a set lesson plan, and thinking about resources. Typically, after new technology was demonstrated, the iCoach would provide follow-up information sessions and support for teachers as they implemented the application using established lesson plans and curriculum with a whole class. But the coaching program had to adapt to the fluidity of the hospital environment, so the hospital setting for her was “a whole different way of looking at things.” This changing environment forced the iCoach to rethink not only how and when she did things but also what was pedagogically sound. For example:

*I wouldn’t normally push to have that many Apps but because the children … are so varied and obviously at different levels, it is good to have Apps for all of those different abilities*. (iCoach-reflection)

Coming from the outside, the iCoach believed she had to spend time establishing relationships and building trust. She wanted the teachers to know not only about her professional role but also details about her experience. Interesting, when asked how teachers could best be guided in the use of mobile technologies, responses from teachers towards the iCoach seemed to be universally positive, such as, *“someone like [the iCoach] who is directed at finding and knowing where to go, in helping us”* (Teacher-011-interview), especially with regard to her skills and ability to support them.

Given the contextual challenges facing the hospital teachers, it became clear that the content focus of coaching had to shift towards identifying time-saving efficiencies to provide teachers instant access to multiple resources from any location and assist them with some of their record-keeping, administrative tasks, and communication needs. One teacher said:

*If I could get my emails directly on an iPad, print out the content wirelessly, be able to email things directly to the school, this would be helpful … it’s so much quicker to use an iPad*. (Teacher-01-interview)

A second critical time-saving efficiency was the introduction of the Dropbox file sharing activity, facilitating instant access to multiple resources from anywhere in the hospital. A teacher reflected on how her teaching practices had changed as a result of the coaching:

*I can access short stories quickly. Most of the poems I need are on the Web instead of trying to find books. It’s made me more confident that I can just turn up and meet the needs of the student…* (Teacher-07-interview)

Meeting the challenge of reluctant teachers led to some surprising outcomes. The iCoach related an anecdote about one of her most reluctant teachers who had told her there was really nothing she could do for her. She explained that this teacher worked with many students and relied heavily on worksheets.

*She’s got this mass of stuff on her shelf — just old faded worksheets are all she’s got – those poor children! She was looking at frogs, and I said “let’s take something that you might use over and over again and let’s try to do something with that.” … My goal is to make lots of iBooks for her so she’ll use them instead of the worksheets*. (iCoach-Interview)

To help the teacher, the iCoach put together an iBook with a link to a frog dissection. The teacher tried it, and saw the students were engaged and afterward started coming to the coach with new ideas for iBooks.

### The Coaching

A unique coaching model emerged that seemed best suited to a setting in which the student population can vary from day to day. It includes the following strategies: individualization by meeting the teachers’ specific needs; goal-setting; modeling practice in small steps; building on success; and coach-facilitated reflection.

#### Individualization

Meeting teachers’ specific needs was a primary consideration for the iCoach to determine issues that were likely to arise when using technology. The iCoach explained that she tried to find something that grabbed teachers’ attention:

*I look for something that [teachers can put] straight into practice … something that they’ll like and find easy to do*. (iCoach-interview)

Sensitive to the unique setting and complex needs hospital teachers faced daily, the iCoach soon realized that coaching required the same amount of individualization as the teaching. She reflected on how differently they worked and how hard she tried to maintain their interest:

*They have a variety of students that come in from all walks of life. They don’t know who they’re going to get, what the students have learnt beforehand … and rather than going full-on from the start, it is about going in gently and just really finding one or two things that spark their interest and can be used straight away and see the results. That’s what will make them want to do it again*. (iCoach-Reflection)

Working with different students and teachers with different needs was challenging, but the iCoach’s knowledge of various Apps was helpful. The iCoach described how, in one day, she worked with a student and teacher on using an App for studying Ancient Rome, another student and teacher on an angles App, and a third child, who was “completely disengaged,” on an App about frog dissections. For her, the goal was always to find an App that would grab the teachers’ attention “to make them see that the technology is worthwhile.” Other Apps like Comic Life, Garage Band and iMovie were all important creative and open-ended apps that the iCoach used to assist in achieving positive outcomes across disciplines. Her aim was to assist the teachers with a collection, or toolbox, of relevant Apps that they could draw on to enhance their teaching.

The importance of goal-setting surfaced in the interviews as an initial strategy in the coaching process. One week the iCoach reported with delight about a small group of teachers who had a clear goal in their mind:

*… Normally I go in and they don’t know what I’m there for. Then I have to provide a lot of guidance to get them to where they’re happy to work with me. All three teachers on Wednesday knew exactly why I was there, had thought about how they wanted to work with me – brilliant!* (iCoach-Reflection)

The importance of modeling, taking small steps, and then building on those successes as a component of the coaching process was illustrated by a teacher who was working with students with head injuries and associated memory issues. He approached the iCoach for advice. Working together, they began to use an App called Snagit to create videos using screen captures. This teacher developed some short presentations about accessibility options for students.

*One of the things I’ve learned with this coaching process is if you find a piece of software that is useful, it works, then you can start to see its potential. It happened with me using the Snagit software. I became better at producing short videos. I’ve had feedback from students about what they think is good so I’ve been reflecting on that and trying to improve …* (Teacher-16-reflection)

The teacher explained that the iCoaching process had made him feel much more confident about using technology “as a first port of call.” He found the technology gave students the opportunity to actively participate in their learning and expressed an ongoing desire to develop a bank of resources accessible to all hospital schoolteachers.

The iCoach perceived the need to implement consecutive sessions to build on concepts over time as one of the more critical components of the coaching process. It led to “aha” moments for teachers to the point where they could see themselves integrating mobile technologies independently. She explained:

*As we’ve gone through different Apps, they’ve been a lot less reluctant to really explore the full capacity of each application … I hear the comments from teachers about “oh wow, I could have done this with this app last week…some child was struggling with the concept and this app just shows it so much more visually*.” (iCoach-Reflection)

A final important strategy that emerged was the reflection process. The iCoach expressed that teachers had been taking part in some thoughtful reflections and she had encouraged them to think about why they used technology and the value it added:

*I think that teachers are not used to reflecting and that’s quite common … Coaching can really transform what they are already doing and the benefits are the engagement that we get from the kids*. (iCoach-Reflection)

As an illustration of the value of reflective practice, one teacher summarized her learning at the conclusion of a four-week coaching cycle:

*Two things I’ll take with me: a huge favorite is Book Creator because you can use that for so many different projects. They can be doing a fiction, a non-fiction, and can put it also into the iMovie. Secondly, being able to Dropbox to the school that day is something I think is so important*. (Teacher-27-reflection)

## Discussion

The goal of this study was to help hospital schoolteachers use technology in an integrated, pedagogical way. After studying the contextual challenges and teachers’ needs over a period of 12 months (McCarthy et al., 2017, 2019), an effective coaching model emerged – a personalized model geared to each teacher and tailored to the unique features of a hospital context. Building a trusting relationship with the hospital teachers and understanding their needs were key components. The teachers saw these components as directly relating to meeting students’ multiple and varied needs while simultaneously collaborating and communicating with their schools. These findings agree with Capurso and Dennis’ (2017) practices that supported the relational and communicational needs of these vulnerable children as part of the European Union project.

The findings from this qualitative study highlight the importance of understanding the context in which coaching will take place for it to be effective and for participants to be receptive. According Knowles, Holton, and Swanson (2015), Penuel, Fishman, Yamaguchi, and Gallagher (2007), and Guskey (2003), consideration must be given to the complex contexts in which teachers work. Saunders (2014) reminds us of the importance of taking into account “the nature and structure of the contexts and to examine any model of professional development in close relation to the systems which influence its design, operation and assessment” (p. 167). In this study, the content and process of coaching were shaped by the contextual influences of the unique hospital setting, where teachers faced complex challenges of providing for a diverse student population at multiple locations requiring impromptu access to curriculum.

Being able to access multiple content areas and multiple types of skills and activities at different levels that are engaging and accessible to different needs instantly is critical for any teacher in this type of situation; yet, this level of expertise, experience, flexibility, innovation, and teaching background is rare. Acknowledging these contextual factors and meeting teachers’ most pressing needs was particularly crucial in providing support to them as they learned to integrate mobile technologies to support their students.

The goal-setting strategy (Elek & Page, 2019) appeared to serve as a turning point where the teachers in the hospital school began fully participating in the process and independently driving the collegial interaction. Once teachers were willing to set their own goals, the coach could assist. The iCoach’s standard professional practice of emailing the teachers after each meeting to reiterate what had been discussed and set a goal for subsequent meetings helped to keep her coaching goal-driven.

An integral part of the coaching was the reflection strategy, which, according to the iCoach, encouraged teachers to question their teaching strategies with the purposeful use of technology. The iCoach prompted teachers at each session to reflect on their current focus and what they had accomplished and then think about what they wanted to expand on the next week, thus restarting the iterative cycle. As a result of these sessions, the teachers began to initiate in an innovative way integration of their pedagogy with appropriate technology.

Many teachers are pressed for time in their daily school settings; in fact, lack of time is generally one of the most commonly voiced teacher concerns and one of the greatest barriers to technology integration in the classroom (Wachira & Keengwe, 2011). However, rather than time, the hospital teachers were more concerned with other issues. A top priority for them was identifying the specific needs of their students and being able to gain immediate access to a wide range of curricula in multiple locations. Therefore, they were more concerned with the accessibility and mobility features of technology (Machado & Chung, 2015). They were also interested in timesaving administrative tools that allowed them to communicate with the enrolled schoolteachers and parents.

In the literature, professional development seems to fail with resistant teachers (Boardman, Argüelles, Vaughn, Hughes, & Klingner, 2005; Jacobs, Boardman, Potvin, & Wang, 2018). That is, it is only successful with those who want to adopt an innovation. Interesting, in this study, even the teachers who were resistant saw some change with our coaching approach. If we view technology as a tool rather than an outcome (Maor & Taylor, 1995), then we can see positive change. For example, even those who were resistant moved from worksheet-driven instruction with no use of technology to integrating technology with more engaging and interactive instruction.

Another important aspect of this model was the willingness on the part of the iCoach to accept flexible learning outcomes from the hospital teachers. She realized that changes in their ability to integrate mobile technologies rather than to create with mobile technologies was a better predictor of their acceptance of more innovative teaching practices.

The reflections of the iCoach and the teachers helped the research team to identify a more effective coaching model. Also significant was the collaboration between the iCoach and the teachers, which that enabled the acceptance of innovations. These changes were also built on the teachers’ willingness and enthusiasm to try out new ideas.

## Conclusion

In conclusion, a set of guiding principles emerged from the research for an effective coaching model, which were later verified and adopted by the leadership group (the principal, three deputy principals, and an IT coordinator). These included:

- Understand and get to know the context within which the teachers work.
- Individualize—Tailor to the context and teachers’ needs (technology creation, use, support).
- Set goals—Identify small reachable goals.
- Practice in small steps; build from success.
- Reflect—what worked, what didn’t, how to move forward?

This study sought to examine how well the model of coaching that emerged met the unique needs of the hospital schoolteachers with a specific focus on the role of the coach. The study illustrated the contextual influences that shaped the coaching model and identified coaching practices that best supported teachers’ individual professional learning needs for successfully integrating technology. Not all coaching is equal; the art of coaching is extremely important. The coaching process was successful because it was individualized and moved each teacher towards a goal of improving teaching for their vulnerable learners using mobile technology as a tool.

### Limitations of the Study

A major limitation of this study was the potential bias the researchers might have brought into the data collection and analysis. To minimize potential researcher bias, we implemented triangulation of data through the use of multiple data sources and independent initial data analysis by two researchers.

The involvement of only one coach in one large hospital school might also limit the generalizability of the findings. However, the model, while it was dependent to some degree on the personality and experience of the iCoach, should be able to be authenticated using the guiding principles.

### Implications for Further Research

Future research should examine whether the next generation of coaches to be trained from within hospital schools can use these guiding principles as effectively as this study suggests. Studies might examine the reactions of teachers to the coaching model and measure the improvement in teachers’ integration of mobile technology in their instruction. To examine the validity of the model and its practical implications, future investigations might also compare our findings to those in other hospital schools and expand the coaching model to other school settings.
